# The positive impact of students in general practice clerkships in rural Germany: diverging views of students, general practitioners, and practice assistants in a prospective survey

**DOI:** 10.1186/s12909-025-07879-0

**Published:** 2025-09-02

**Authors:** Wolfgang Blank, Anna Sporkert, Lars Daenenfaust, Sabine Gehrke-Beck

**Affiliations:** 1 General Practice “Im Bayerwald”, Am Alten Sportplatz 3, 94259 Kirchberg im Wald, Germany; 2 Danube Private University GmbH (DPU), Steiner Landstraße 124, , Krems an der Donau, 3500 Austria; 3 Die LandArztMacher GbR, Am Alten Sportplatz 3, 94259 Kirchberg im Wald, Germany; 4 Institute for Health Economics and Clinical Epidemiology (IGKE), University Hospital Cologne, Gleueler Str. 176-178, 50935 Köln, Germany; 5 Institute for General Practice, Charité-Universitätsmedizin Berlin, Corporate Member of Freie Universität Berlin and Humboldt-Universität zu Berlin, Charité Campus Mitte, Charitéplatz 1, 10117 Berlin, Germany

**Keywords:** General practice, Medical education, Clinical clerkship, Health care team, Motivation

## Abstract

**Background:**

Recruiting and retention of teaching practices is challenging. Although GP teaching physicians are predominantly intrinsically motivated, the time required for teaching is often cited as a barrier. Practice teams could benefit, if the students’ learning in practice is optimized in such a way that there is also a noticeable positive impact in daily work. To inform this, we explored students’, general practitioners’ (GPs) and practice assistants’ (PAs) views on the positive impact that students could have during a general practice clerkship (GPC). We asked participants and teaching practices of the “Excellent Summer”, a GPC project in rural Bavaria.

**Methods:**

Based on a qualitative survey, we developed a questionnaire with 12 items rating the potential benefits of students in practice. Students, GPs and PAs gave their subjective assessment of the benefits on a 4-point Likert scale. The students completed the questionnaire before and after their clinical traineeship. The assessments of the three groups were compared using variance analysis, the pre-post assessment among students using the Wilcoxon signed-rank test.

**Results:**

44 students, 59 GPs and 29 PAs (100% response rate) responded. Initially, the students rated their support in routine tasks and their influence on cultural attitudes higher than GPS and PAs. GPs and PAs rated students´contribution through up-to-date knowledge and the stimulation of self-reflection higher than students themselves. They similarly saw enjoyment with teaching and sharing experiences as bigger than students. MFAs perceived the influence of students in a similar way to the GPs, but saw the benefit of students for patient care more positively. During the clinical traineeship, the students’ assessment approached that of the practice team in some aspects.

**Conclusions:**

Differing expectations can be addressed with customized preparation and instructions for students and practice teams and optimize benefits of students´ activities. Positive impact of students´ clerkships can facilitate recruiting and retainment of teaching practices.

**Supplementary Information:**

The online version contains supplementary material available at 10.1186/s12909-025-07879-0.

## Introduction

Teaching in general practice is being expanded internationally to secure the next generation of general practitioners (GPs) [1–3]. Strengthening primary care teaching is also being demanded and discussed in Germany [4, 5]. With an increasing demand of student placements in General Practice, recruitment and retention of out-patient teaching practices is becoming more challenging and difficulties in supply of adequate number and quality of community teaching are discussed internationally [6–8].

Currently, clinical learning experiences in general practice in Germany are limited to a compulsory General Practice Clerkship (GPC) of 4 weeks and a clinical placement of 2 weeks at most faculties. Students can choose to spend part of their practical year in general practice but only a small number of students choose this option. Some faculties offer additional clinical experience in outpatient care.

Therefore, supply of adequate number of teaching practices is not a major concern, but retention and ongoing engagement of current teaching practices is threatened by increasing clinical and administrative workload [9]. This is particularly true in underserved regions where many practitioners are retiring [10]. High patient volumes, short consultation times and administrative tasks associated with self-employment and practice organization may lead to GPs quitting teaching tasks. In rural areas, work load and undersupply of GPs limit student placements, while at the same time there is a high need to gain young doctors willing to work in the area by offering local student placements [11].

The motivation of GPs to teach is well researched. Intrinsic motivation is predominant: wanting to pass on knowledge, supporting the recruiting of young doctors, and benefits for their own knowledge are most important factors. Financial and nonfinancial compensation (like free continuing medical education or access to databases and literature) play a minor role [8–16]. Problems with rooming students and time expenditure are main barriers for accepting students in practices [12–15, 17, 18].

To balance student education and patient care, it would be helpful to integrate students in daily work and generate benefits for daily work and practice teams [19, 20]. The concept of added-value in medical education describes the learning of students in clinical settings as a win-win-win situation with added value for patient care, student´s learning and the health care system [21–23]. The perception of added value has been looked at in the perspective of students, showing that students estimate that they could contribute to patient care and seem willing to do so, although some prefer to concentrate on learning [24, 25]. The perspective of practice teams on students´ contribution and positive impact in practice settings are lacking.

Research on the concept of students adding value to patient care has been conducted in countries with a more prevalent teaching of general practice and different healthcare systems. In Germany, GPCs are not very structured and neither students nor practice teams are supported or prepared to facilitate and enhance learning in this context. There are no defined instructions or suggestions for suitable learning goals or activities. Lack of structure was found to hinder integration of students in the practice setting [26]. Projects addressing the need for more guidance mainly focused on learning outcomes of students and not considering outcomes for patient care and practice teams [27]. To develop a customized preparation and guidance for students and teachers that also looks at possible benefits of students´ presence, we explored the perception of positive impact of students in GP practice settings. Looking at the positive impact of students in the practice environment and patient care could inform support and preparation for students and practice teams with guidance and encouragement for learning activities that are valuable for both learning od students and everyday work of practice teams. More effective and time efficient student supervision in practice placements and GPCs can encourage the recruiting and retainment of teaching practices.

## Methods

### Aim and setting

Since 2014, the “Excellent Summer” project offers GPC placements to students in rural Bavaria to improve future workforce in the region. The GPC is accompanied by additional teaching sessions and team building as well as free accommodation for students. While the project has been shown to improve attitudes to working in rural regions and number of students interested in participating were increasing [28], finding new teaching practices became difficult in the last years.

To look at the perception of students´ positive impact from both students´ and practice teams´ perspectives, we surveyed the students, supervising doctors and PAs during GPCs in the summer 2024.

### Development of questionnaire

We didn´t find a validated measure for measuring attitudes to or beliefs of students´ presence benefits in GP practices and therefor developed suitable items with a qualitative survey.

An online survey with open questions was sent to all participants (*n* = 43) of the Excellent Summer project in 2022. Students were asked to answer the question “What benefit did the practice have form your clerkship?” and could freely formulate their ideas. All participants answered the online survey. Their answers were analyzed with qualitative content analysis after Mayring [29]. Two practical year students and two residents initially summarized and categorized responses individually and then discussed findings with WB. We derived 12 separate perceived advantages, that covered practical, educational, and social aspects of working together in the practice (see suppl. 1). We formulated 12 questions to ask for the perception of student’s impact considering these advantages, using a 4-point Likert scale for rating. Questions to collect sociodemographic data as well as the previous teaching experience (for GPs) and study year and clerkship experience (for students) were added. The questionnaire was piloted from September to November 2023 with five practical year students. With their feedback, some questions were revised for better comprehension and clarity.

### Data collection

The final questionnaire was distributed as an online survey via umfragenonline.com to all students participating in the Excellent Summer Project 2024. As GPC students in Germany are mostly not exposed to GP practice settings in their studies before the GPC, we assumed that perspectives will change with the GPC experience and surveyed students before and after their GPC, allowing for pre-post comparisons. The survey was sent at the start of the first week of the GCP and the end of the last week of the GCP respectively. Up to three automated reminders were sent to those that didn't respond yet.

As most teaching practices were familiar to supervise and teach students, we did not expect major changes with one specific GPC and distributed the survey to all GPs and PAs in the teaching practices after the GPCs only.

As not all GPs and PAs had an email account, the questionnaire was distributed in paper form via the students. Students were given questionnaires for all supervising doctors in a practice (1–2 doctors) and for PAs if they were involved in student supervision and passed on the questionnaires as well as envelopes. The questionnaires were returned anonymously in a ballot box.

### Data analysis

Statistical analyses were performed on an exploratory basis. After checking the accuracy of data, the Wilcoxon signed-rank test was used to compare the pre-post changes among students. We then compared the replies among the three groups by applying chi-square tests without post-hoc between-group comparisons. In this analysis, the ‘post’ values from students were entered. A p value of less than 0.05 was considered significant.

## Results

Of 44 students participating in the GPC project, 44 answered the questionnaire (response rate 100%). The students (79% females, mean age 24 years) were in their first (9%), second (50%), third (30%), or higher year (11%) of their medical education. The GPC was the first, second, third, fourth or fifth clerkship for 21%, 41%, 21%, 14%, and 5%, respectively. Students handed out 86 questionnaires in their practices and all 88 questionnaires were returned to the ballot box. 59 GPs (32% females, 59% males, rest not specified) answered the survey. and 29 PAs (85% females, mean age 39 years) answered the survey. Of the responding GPs, 36 (67%) owned their practice, while 14 (26%) w

ere employed and 4 (7%) were in training. The GPs’ mean age was 48 years (range 27 to 69, data missing for 5) and most of them were well-experienced with teaching students in their practice.

The three groups had differing views on the students’ impact. The views of GPs and PAs were largely similar, but students’ views (before and after their GPC) differed in several aspects (Table 1). GPs saw most benefit in their own enjoyment of teaching and sharing their enthusiasm for patient care, as well as the positive atmosphere students bring. They also highly valued the up-to-date knowledge and the inspiration for self-reflection that students contribute. Students also rated these aspects positively, but saw lower benefit of their contribution of current knowledge and the self-reflection they can stimulate (item 3 and 4). In addition, students gave lower ratings to the aspect that GPs feel inspired by sharing their work experience with students and enjoy teaching is (items 10 and 11). GPs and PAs saw a bigger advantage in students’ perception and feedback of practice processes and their help to optimize teamwork than students themselves (item 5). GPs and PAs also felt that team spirit and mood strongly benefitted, while students only saw a moderate influence (item 9). These differences were moderate in size (range from 0.4 to 0.6 on the 1 to 4 scale), but statistically significant. In addition, students saw a slightly greater benefit than GPs in the additional time they can spend with patients (item 7).

**Table 1:** Students’, gps‘, and pas’ views on the impact of the students

	Students (*n* = 44)	GPs (*n* = 59)	PAs (*n* = 29)	*P* value (chi-square)
*Students*				
1. Relieve the team in routine tasks	2.9 ± 0.8	2.7 ± 1.0	3.0 ± 0.8	
	1/13/21/9	8/18/16/17	1/5/15/8	0.08
**2. Relieve the team in medical patient care**	2.9 ± 0.7	2.9 ± 1.0	**3.3 ± 0.6**	
	2/8/28/6	6/13/22/17	0/2/15/12	0.020*
**3. Bring current knowledge**	**2.8 ± 0.8**	3.5 ± 0.6	3.4 ± 0.7	
	2/13/23/6	0/4/22/32	0/3/12/14	< 0.001*
**4. Stimulate self-reflection of the GP**	**2.9 ± 0.9**	3.5 ± 0.7	3.4 ± 0.8	
	3/10/20/11	1/3/18/36	1/2/11/15	0.006*
**5. Rethink processes in the practice**	**2.7 ± 0.7**	3.2 ± 0.8	3.3 ± 0.7	
	1/15/23/5	1/11/21/24	0/3/15/11	0.018*
6. Question traditional attitudes	2.9 ± 0.8	2.8 ± 1.0	3.0 ± 1.0	
	1/12/19/11	8/12/22/15	3/4/11/10	0.42
7. Can take more time for patients	3.6 ± 0.5	3.3 ± 0.8	3,6 ± 0.6	
	0/1/15/28	2/7/24/26	0/1/10/18	0.17
8. Convey new unbiased aspects to individual patients	2.9 ± 0.7	3.1 ± 0.8	3.4 ± 0.8	
	2/7/27/8	3/7/30/19	1/3/9/16	0.08
**9. Bring a positive atmosphere into the team**	**3.4 ± 0.6**	3.6 ± 0.7	3.7 ± 0.5	
	0/3/21/20	2/0/18/39	0/1/6/23	0.030*
*GPs*				
**10. Enjoy showing students their own work**	**3.4 ± 0.6**	3.8 ± 0.4	3.8 ± 0.4	
	0/1/23/20	0/0/11/48	0/0/6/24	0.001*
**11. Find teaching appealing**	**3.3 ± 0.6**	3.8 ± 0.4	3.8 ± 0.4	
	0/3/26/15	0/0/15/44	0/0/7/23	< 0.001*
*Patients*				
12. Appreciate that trainees are recruited to the region	3.4 ± 0.7	3.5 ± 0.6	3.7 ± 0.5	
	1/3/19/21	0/3/26/30	0/0/10/20	0.44

Data are given a means ± standard deviations followed by counts; scale from 1 “Disagree” to 4 “Agree”; *n* = 132; data completeness over 96%; differences highlighted by bold print

PAs perceived most aspects similar to GPs, but rated the students’ contribution to patient care more positively. They saw the benefit of the support they bring to medical patient care (item 2) and their additional time for patients (item 7) more positive than GPs. They valued the later aspect in the same way as the students, whereas GPs were less likely to expect benefit from the additional time students devote to patient care. (item 7).

Table 2 shows how the students’ views changed between the start and the end of their GPC. Compared to the perception of GPs and PAs, students initially estimated their support in routine tasks, e.g. taking blood samples, higher than after their GPC (Table 2, item 1). Very similarly, students initially also perceived their influence on cultural aspects (item 6) as bigger as after the GPC. On the other hand, students´ ratings of GPs enjoyment with showing their work increased after the GPC. (Item 10). Although the students´ views changed with the GPC, they still rated this aspect lower than the GPs themselves.

**Table 2:** Students‘ views on their impact as elicited before and after their GPC

Students	Before	After	Higher/same/ lower	*P* value (sign test)
**1. Relieve the team in routine tasks**	**3.3 ± 0.6**	**2.9 ± 0.8**	**7 / 17 / 20**	**0.019**
2. Relieve the team in medical patient care	2.7 ± 0.5	2.9 ± 0.7	16 / 20 / 8	0.15
3. Bring current knowledge	2.7 ± 0.9	2.8 ± 0.8	13 / 20 / 11	0.84
4. Stimulate self-reflection of GPs	2.9 ± 0.8	2.9 ± 0.9	11 / 20 / 13	0.84
5. Rethink processes in the practice	2,9 ± 0.7	2.7 ± 0.7	7 / 22 / 15	0.13
**6. Question traditional attitudes**	**3.3 ± 0.7**	**2.9 ± 0.8**	**7 / 18 / 18**	**0.043**
7. Can take more time for patients	3.5 ± 0.6	3.6 ± 0.5	11 / 26 / 6	0.33
8. Convey new unbiased aspects to individual patients	3.1 ± 0.7	2.9 ± 0.7	11 / 19 / 14	0.69
9. Bring a positive atmosphere into the team	3.2 ± 0.6	3.4 ± 0.6	12 / 26 / 6	0.24
*GPs*				
10. Enjoy showing students their own work	3.2 ± 0.7	3.4 ± 0.6	16 / 22 / 6	0.052
11. Find teaching appealing	3.2 ± 0.7	3.3 ± 0.6	13 / 22 / 9	0.52
*Patients*				
12. Appreciate that trainees are recruited to the region	3.3 ± 0.7	3.4 ± 0.7	9 / 29 / 6	0.61

Data are given a means ± standard deviations followed by change counts; scale from 1 “Disagree” to 4 “Agree”; *n* = 44; significant changes marked by asterisks and bold print

## Discussion

In summary, all groups surveyed saw benefits from students in practices, but students rated their beneficial contributions to patient care higher than GPs and GPs saw a greater benefit from the knowledge and new perspectives that students bring with them and the enjoyment derived from teaching for themselves and the practice team. PAs saw students’ presence comparably positive and rated their contributions to patient care more positively.

### Students in patient care

Students seem to expect to be helpful in medical patient care with a trend towards feeling less useful by the end of their practice placement. This positive expectation to support the practice team should be used, even if differentiation from auxiliary work or “scutwork” is important [21, 30]. Which tasks are suitable for students’ learning and not a mere delegation of unpopular auxiliary tasks and how a differentiation between both can be achieved has already been discussed and proposed for emergency departments [30]. From the student’s perspective, willingness to contribute to daily work has been shown, but motivation is highest for tasks that are the future duties of doctors and the main aim of learning needs to be kept in mind [21, 24, 25, 30]. Tasks, that have both the goal of learning and of contributing to care have been proposed [20, 22, 30], but need to be adapted to specific practice settings. In general practice, students could see patients with common problems like backaches and upper respiratory infections and perform health checks with both the opportunity to learn and to support patient care.

As GP work load and time resources are a major concern, instruction and integration of students in practice working routines should be conceived as a team task [20, 22]. PAs have a positive perception of students' usefulness in patient care and are involved in the relevant practice processes. They can be helpfully involved in students’ instruction and supervision. They have the first contact with patients and can identify suitable patients and can also explain practice procedures well to students.

### Students as teachers

GPs value the up-to-date knowledge that students bring along in their practices and the questions they ask to stimulate reflection on established procedures and assumptions. It is well known, that knowledge from students is a motivation for GPs to teach [12–18, 31]. Clinical teaching can take both directions and students “teach” their teachers as well [32]. Students themselves seem less confident in their role as teachers for experienced GPs and practice staff. Students’ knowledge as well as the questions they ask could be used in a more targeted way. Students could be encouraged to prepare short inputs or team education for the practice team. Case discussions and journal clubs are educational formats that enable exchange and knowledge transfer. Difficult patient cases that raise question for students can be reflected on by the whole team. Didactic qualification for GPs in teaching practices could include suggestions for these options of knowledge transfer and should encourage using reflection as part of being a GP as well as in the role of a teacher.

### Students as change agents

Although the practice teams rate students’ stimulation of reflection related to patient care and overthinking processes in the practice (item 4 and 5) positively, students influence on the teams’ attitudes are seen with more doubt. Students can have a role as change agents in faculty as well as health care teams and professional identity formation can include assuming a role as a change agents [33–35]. Students’ ability to look at teams, processes and systems from an outsider perspective can bring on ideas for change and practice teams open for new ideas and change can profit from that. Actively seeking input for practice and team organization could easily be done in team meetings or at the end of a GPC. While most didactic qualifications for GP teachers include instructions to give regular and helpful feedback to students, actively seeking feedback from students could be promoted just as well.

### Strength and limitations

To our knowledge, this study is the first to examine the benefits of student presence and learning in general practice from the perspectives of both students and practice teams. PAs are involved in student supervision and teaching, but rarely included in research on learning in practice; their perspective was specifically explored in this study. Only 27 PAs were involved in teaching and supervising students, so only PAs that are interested in accepting and supervising students have responded. Possibly, not all PAs are interested teaching and ready to be involved in student supervision, so these results apply only to those willing to do so. We developed a survey from students’ experiences to ensure that the items were meaningful to the setting and we had a high response rate. We did not explore GPs and PAs views for the development of the questionnaire items to limit time expenditure for practice teams and as we considered students perspective more important at this stage. A limitation is that the survey is monocentric and the results are not necessarily generalizable to different settings, such as urban practices, the hospital setting, or other countries with different health care systems. The survey did not collect objective measures of benefits as time saving or patient satisfaction but explores the views of students and practice teams involved.

## Conclusion

Both Students and practice teams perceived multiple benefits of student GPCs, although there were differing views on some aspects. We did not explore students’ views on the positive learning outcomes of tasks and activities that are beneficial to practice teams. Further research should address the implementation of targeted student activities that enhance the benefits to the practice, but at the same time investigate learning outcomes.

For GPCs or other clinical placements in teaching practices, the findings suggest to address differing views on students’ activities before the GPC and provide better preparation and suggestions for beneficial learning activities. Practice team can be encouraged to involve students in patient care and students can be encouraged to share their knowledge and discuss patient care to promote reflection as well as to provide feed-back to practice organization. Improving patient care and practice routines while maintaining high learning outcomes should be addressed in didactic qualifications for GP practice teams and in preparatory workshops or written manuals for students. The positive impact of students in teaching practices could help to retain teaching practices despite increasing workloads and help to recruit new practices.

Students' contribution to patient care and benefits to practice teams may be particularly helpful in rural areas with a low density of doctors and high patient numbers. With this, the training of students offers both the opportunity to support and enrich practice teams and the chance to recruit future workforce in the region.

## Supplementary Information

Below is the link to the electronic supplementary material.

Supplementary Material 1.

## Data Availability

The datasets used and analysed during the current study are available from the corresponding author on reasonable request.

## Abbreviations

GP: General practitioner
GPC: General practice clerkship
PA: Practice assistant
