# Supplementary material for: The positive impact of students in general practice clerkships in rural Germany: diverging views of students, general practitioners, and practice assistants in a prospective survey
Source: BMC Med Educ. 2025 Sep 2;25:1243. doi: 10.1186/s12909-025-07879-0 (PMC12403355; doi:10.1186/s12909-025-07879-0)
Supplement: Supplementary file 1 — Supplementary Material 1. [file 12909_2025_7879_MOESM1_ESM.docx]

**Additional file 1 Items derived for the questionnaire**

**Aspects of students positive impact**

**1.** **Students noticeably relieve the team of routine tasks.**

Students carry out routine tasks such as taking blood samples, inserting needles, attaching infusions, running errands, making phone calls, and filling out information sheets. This relieves the team of routine tasks

**2. Students noticeably relieve the team in medical patient care.**

Students can (after sufficient training) carry out history taking and clinical examinations, document them and present the patients. This relieves the team of routine medical tasks.

**3. Students bring current knowledge from the university into the daily work routine by asking questions and providing feedback.**

Through discussions during and after patient presentations, students contribute their knowledge from university training.

**4. Through their questions about illnesses, examinations and treatments, students can encourage the doctors in the team to reflect on their skills and optimize them if necessary.**

Discussing patients with students can encourage them to reflect on their own medical approach Routines or thought patterns (“I've always done it this way”) are thus questioned and can be put to the test.

5. **As new employees, students can perceive and react to practice processes and reflect on them without bias. This can help to optimize teamwork.**

Through their experiences in various other areas (e.g. clinical clerkship, clinic, block clerkship in other institutions), students have experienced different procedures for the same diseases and can provide constructive feedback on how these were implemented elsewhere. This can possibly lead to optimization possibilities for their own area.

**6. Students can use their attitude to life and their own and their own metropolitan horizon of experience, they can question traditional attitudes and perspectives in the team (e.g., racism, sexism, diversity).**

Students can give feedback to the team, if they perceive discriminatory behavior or thinking in patients or in the team.

**7. Students can take more time for patients and relatives and give them the feeling that they have been well looked after.**

By taking detailed medical histories of patients and relatives, students get to know their illnesses and complaints better, even in longer conversations, to the benefit of both sides. Patients can thus feel that they are in better hands than usual in the practice/clinic.

**8. Students can convey new aspects of individual patients to the team because they can perceive them without being biased by their previous history.**

As students care for patients completely unbiased by previous experience, new aspects can arise with regard to the medical history and diagnostics that may no longer be perceived by the team due to the many years of care.

**9. Students bring a positive atmosphere to the team.**

Through their motivation and commitment, students bring a breath of fresh air to the team. The change from the other working days can have a positive effect.

**10. GPs enjoy showing students their own work and sharing their enthusiasm for patient care.**

**and share their enthusiasm for patient care.**

Their own enjoyment of and satisfaction with their daily work can be shared with committed students

**11. Training students and putting teaching into practice is appealing for teachers.**

The increasing learning success of students in their own field of activity first hand is a pleasure for teachers.

**12. Patients greatly appreciate the fact that students are being trained and that potential young talent is being recruited for the region.**

Patients and the local area perceive it positively when teaching is carried out in addition to patient care and there is a commitment to attracting young doctors to the region
